# Molecular Cloning and Expression Analysis of IgD in Nile Tilapia (*Oreochromis niloticus*) in Response to *Streptococcus agalactiae* Stimulus

**DOI:** 10.3390/ijms17030348

**Published:** 2016-03-08

**Authors:** Bei Wang, Pei Wang, Zao-He Wu, Yi-Shan Lu, Zhong-Liang Wang, Ji-Chang Jian

**Affiliations:** 1 College of Fishery, Guangdong Ocean University, Guangdong Provincial Key Laboratory of Pathogenic Biology and Epidemiology for Aquatic Economic Animals, Guangdong Key Laboratory of Control for Diseases of Aquatic Economic Animals, Zhanjiang 524088, China; wong19820204@126.com (B.W.); wuzaohe@163.com (Z.-H.W.); fishdis@163.com (Y.-S.L.); leong2006@126.com (Z.-L.W.); 2 Guangxi Key Laboratory of Beibu Gulf Marine Biodiversity Conservation, Qinzhou 535099, China; wangpei1108@163.com

**Keywords:** *Oreochromis niloticus*, IgD, *Streptococcus agalactiae*, fluorescent quantitative real-time PCR

## Abstract

IgD is considered to be a recently-evolved Ig and a puzzling molecule, being previously found in all vertebrate taxa, except for birds. Although IgD likely plays an important role in vertebrate immune responses, the function of IgD in Nile tilapia (*Oreochromis niloticus*) is virtually unknown. In the present study, a membrane form of IgD (mIgD) heavy chains were cloned from the GIFT strain of Nile tilapia (designated *On-mIgD*). The *On-mIgD* heavy chain’s cDNA is composed of 3347 bp with a 31 bp of 5′-UTR, 3015 bp open reading frame (ORF) and 301 bp 3′-UTR, encoding a polypeptide of 1004 amino acids (GenBank accession no: KF530821). Phylogenetic analysis revealed that *On-mIgD* heavy chains showed the highest similarity to *Siniperca chuatsi*. Quantitative real-time PCR (qRT-PCR) analysis showed that *On-mIgD* expression occurred predominately in head kidney, thymus, spleen, and kidney. After *Streptococcus agalactiae* infection, transcripts of *On-mIgD* increased and reached its peak at 48 h in the head kidney and thymus, and 72 h in the spleen, respectively. Taken together, these results collectively indicated that IgD could possibly have a key role to play in the immune response when bacterial infections in Nile tilapia.

## 1. Introduction

It is well-known that two heavy chains and two light chains consisting of the Igs (immunoglobulins), which are important effective molecules in humoral immunity of primates and rodents. Whereas Igs molecules divide into five classes in primates and rodents, not all five types of Igs exist in all species of fish. Different from the IgM, which was the only Ig molecule found to be common to all species of fish, the IgD gene has been only shown to be present in some fish species [1,2,3,4,5,6,7]. Previous studies show that IgD is an ancient Ig molecule, so identification of the *IgD* gene in teleost contributed to changes in the evolutionary view of IgD. Spleen, head kidney, kidney, thymus, and gut comprise the major lymphoid organs of fish, yet bone marrow and lymph nodes constitute the immune organs of mammals. The fish immune system is far simpler than that of primates and rodents [8]. It is necessary to analyze IgD in teleosts, as it will provide a reference for further research in this field.

The structures of fish IgD genes have a magical heavy chain, which contain a large amount of constant domains compared with mammals. Humans [9] and mice [10], have only three and two domains found in IgD, respectively. Additionally.in the teleost IgD, the hinge region is missing. Further, IgD gene structures of teleosts exhibit high genetic diversity. For example, a duplication of domains δ2-δ3-δ4 are found in salmon [2], halibut (*Hippoglossus hippoglossus*) [11], and carfish (*Ictalurus punctatus*) [12], but not found in flounder [4]. Interestingly, two different forms of mammals’ IgD rely on two distinct forms of existence: secreted and membrane-bound forms. These two forms are distinguished primarily by the amino acid sequence at the carboxyl terminus of the heavy chain [10]. Although the secretory type of IgD has so far been reported in catfish (*Ictalurus punctatus*) [13] and rainbow trout (*Oncorhynchus mykiss*) [7], to our knowledge the information regarding the function of membrane IgD in fish is insufficient.

Nile tilapia (*Oreochromis niloticus*) is a vital commercial fish and cultured all over the world, particularly in the Guangdong province, southern China. Moreover, it also generally exists in brackish water in estuaries around the world [14]. Due to *O. niloticus’* (*Oreochromis niloticus*) rapid growth and hardiness, it is considered to be a good species for aquaculture. The infection of *Streptococcus agalactiae* (*S. agalactiae*) in epidemic proportions can cause severe economic losses in wild and cultured hybrid tilapia (*Oreochromis niloticus × Oreochromis aureus*) worldwide [15,16,17]. However, the information about the IgD of *O. niloticus* is largely unknown. Study on the structures and distribution of the *IgD* gene in tilapia’s different organs will enrich our knowledge of the fish immune system. In the present study, we cloned a membrane form of the *IgD* gene from Nile tilapia (*O. niloticus*), subsequently investigated its tissue distribution and expression characteristics in response to *S. agalactiae* infection. These findings clearly give more information for the immunoglobulin class diversity in the teleost fish, and suggest that IgD in tilapia plays an important role in innate immune response.

## 2. Results

### 2.1. Characterization of On-mIgD

**Figure 1:**
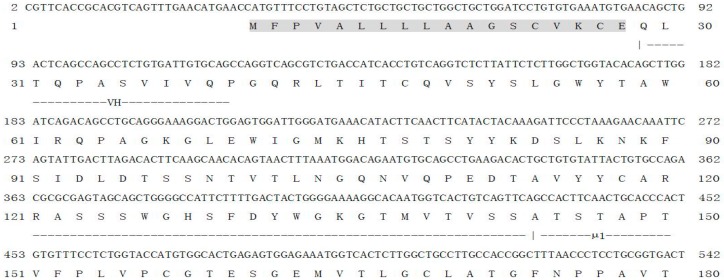

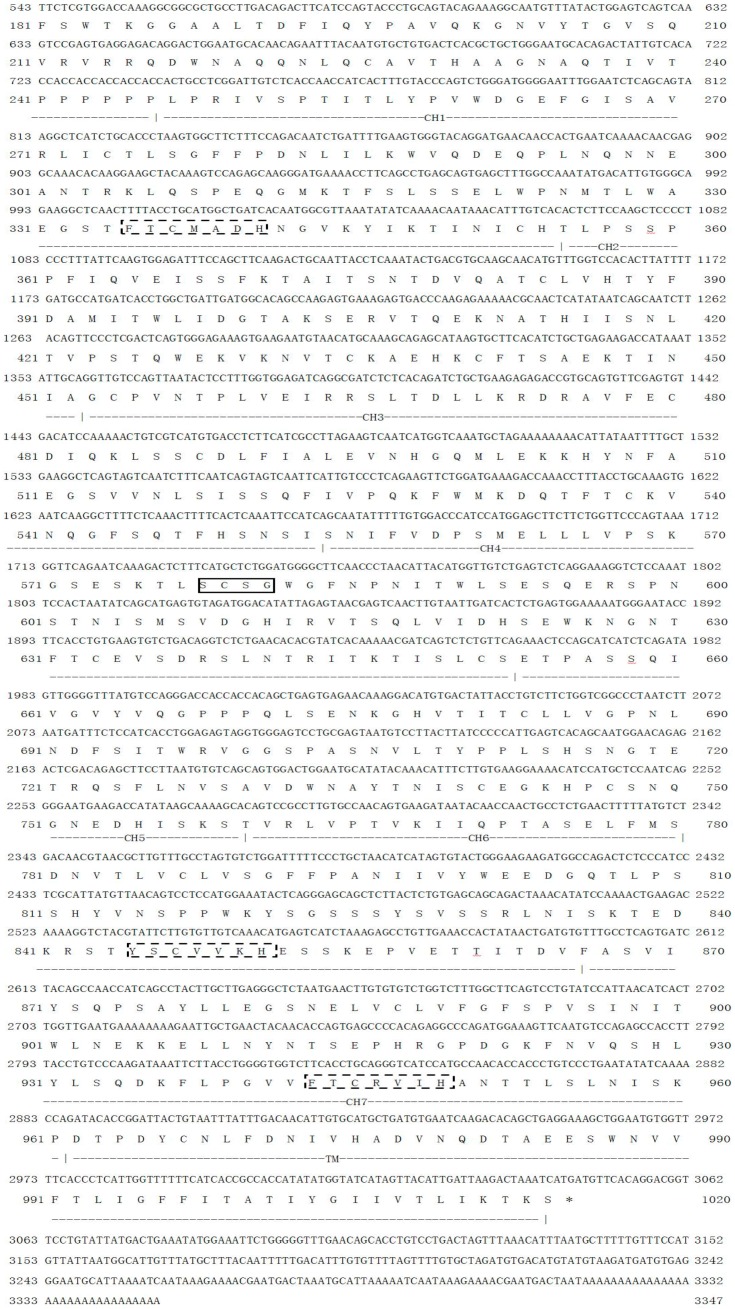
Nucleotide and amino acid sequence of Nile tilapia *mIgD* cDNA (GenBank Accession No. KF530821). Use the numbers on the left and right to show the nucleotide positions. The stop codon is represented with an asterisk (*). The polyadenylation signal is shown as underlined. The signal peptide domain, glycosaminoglycan attachment domain, and major histocompatibility complex proteins domain are shown as shaded, plain and dashed boxes.

**Figure 2:**
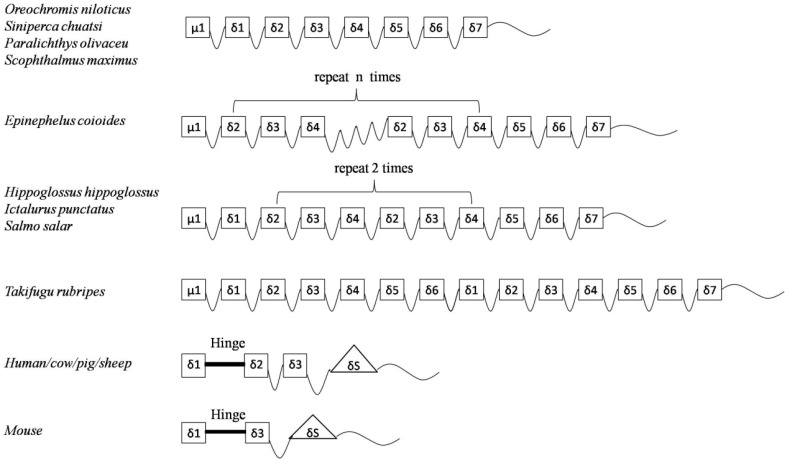
Structural schematic of the IgD constant region included in teleost, primates, rodents, and artiodactyls.

The full length of On-mIgD cDNA (GenBank ID: KF530821) contained a 5′ untranslated region (UTR) of 31 bp, a 3′-UTR of 301 bp with two typical polyadenylation signals (AATAAA), and the open reading frame (ORF) includes 3015 nucleobases, their sequence encoding a protein that include 1004 amino acids. Software algorithms show the molecular weight and theoretical isoelectric point of the protein is 110.9 and 6.24 kDa, respectively. The deduced amino acid sequence of On-mIgD had a glycosaminoglycan attachment site (SGWG) and major histocompatibility complex protein sites (FTCMADH, YSCVVKH, and FTCRVIH) (Figure 1). The structure of Nile tilapia (*O. niloticus*) IgD contained the variable (V), diversity (D) and joining (J) region, the constant region consists of a μ1 domain, 7 CH domains (CH1-CH2-CH3-CH4-CH5-CH6-CH7) and transmembrane regions (Figure 2). However, IgD of mammals use a hinge region segment instead of ancestral δ molecule (Figure 2).

### 2.2. Homology Search

**Figure 3:**
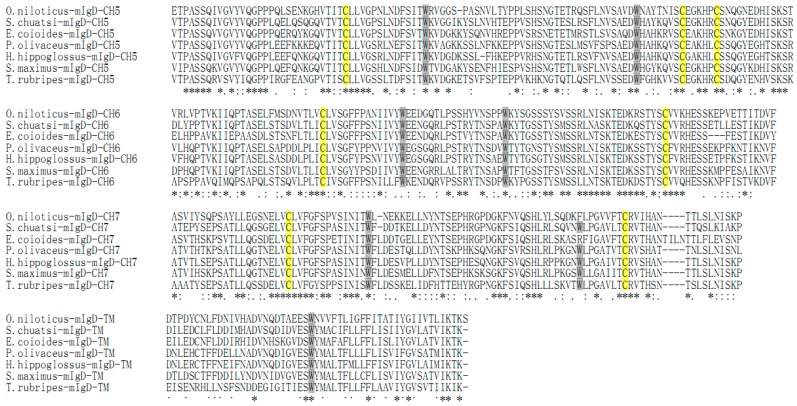

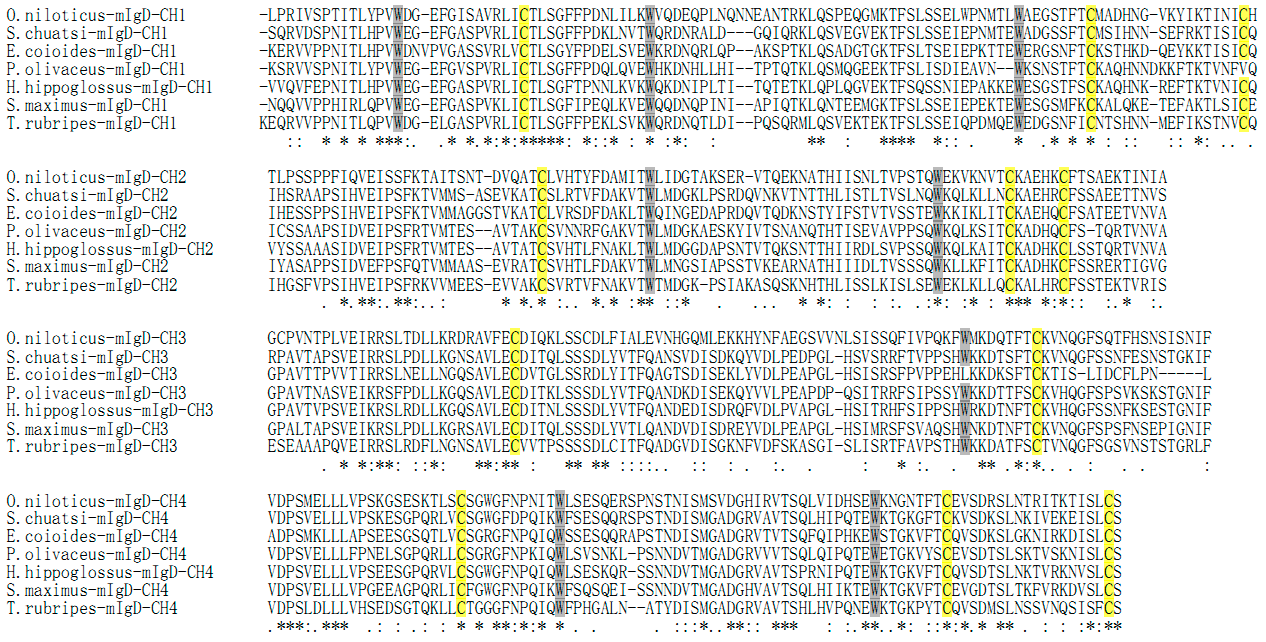
Multiple alignments of the On-mIgD amino acid sequence with other known On-mIgD. Identical residues are indicated with asterisks (*), and similar amino acids are indicated with dots and colons. The conserved cysteine residues predicted important for disulphide linkage are yellow highlight. Tryptophan residues required for the formation and stabilization of the tertiary structure of immunoglobulin are indicated by shades of grey. GenBank accession numbers are as follow: *O. niloticus* KF530821; *S. chuatsi* ACO88906.1; *E. coioides* AFI33218.1; *P. olivaceus* BAB41204.1; *H. hippoglossus* AAL79933.1; *S. maximus* AFQ38975.1; *T. rubripes* BAD34541.1.

**Figure 4:**
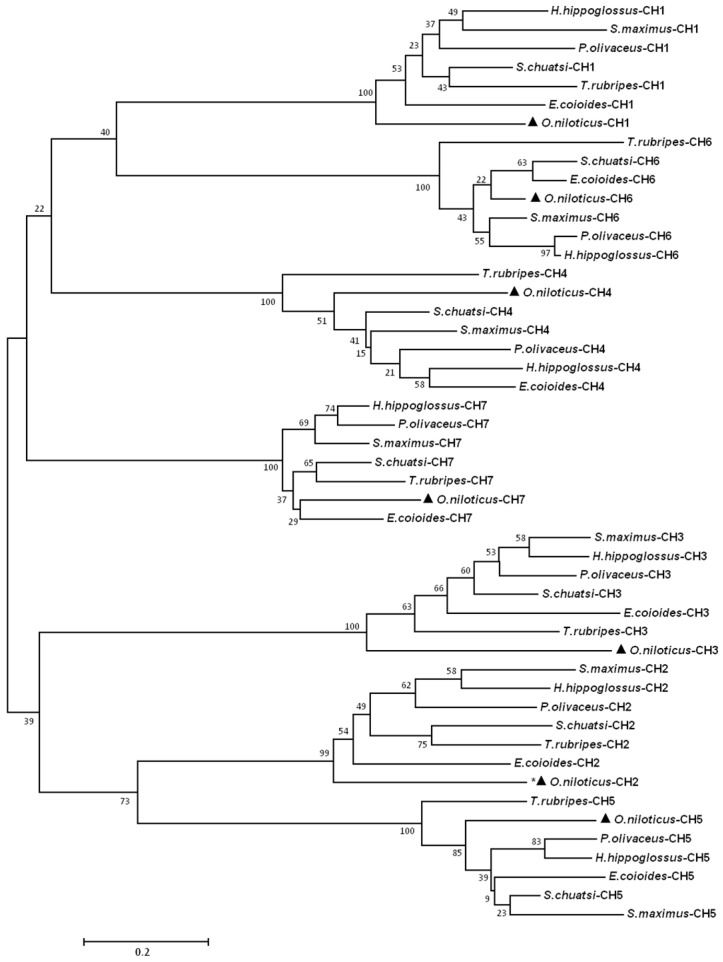
Phylogenetic analysis of IgD CH domains in teleost by using the Neighbor-Joining method. Bootstrap values are indicated at nodes. The IgD CH domains of Nile tilapia are indicated with black triangles. The CH2 domain of Nile tilapia is clustered closely with CH5 indicated with asterisks (*). GenBank accession numbers are as follows: *O. niloticus* KF530821; *S. chuatsi* ACO88906.1; *E. coioides* AFI33218.1; *P. olivaceus* BAB41204.1; *H. hippoglossus* AAL79933.1; *S. maximus* AFQ38975.1; *T. rubripes* BAD34541.1.

Alignment of the seven species of fish amino acid mIgD sequences revealed that all CH domains of mIgD are rather conserved in terms of cysteine residues involved in the formation of inter-domain and inter-domain disulfide bonds (Figure 3). After the first and second cysteine, there are 11–14 and eight tryptophan residue positions conserved and located, respectively. In δ3, the first tryptophan residue is replaced by another amino acid in teleosts. The On-mIgD domains were found to show the highest identity to those of freshwater grouper (50%–76%), followed by 50%–70% and 48%–70% identity to those of orange-spotted grouper and turbot, respectively. The CH7 domain was found to be the most conserved domain among fish species. A phylogenetic tree based on teleost IgD heavy chain constant regions generated by the neighbor-joining method suggests that all teleosts cluster together, and that CH1 is clustered closely with CH6, and CH2 is clustered closely with CH5, respectively (Figure 4).

### 2.3. Tissue-Specific Expression of On-mIgD mRNA

**Figure 5:**
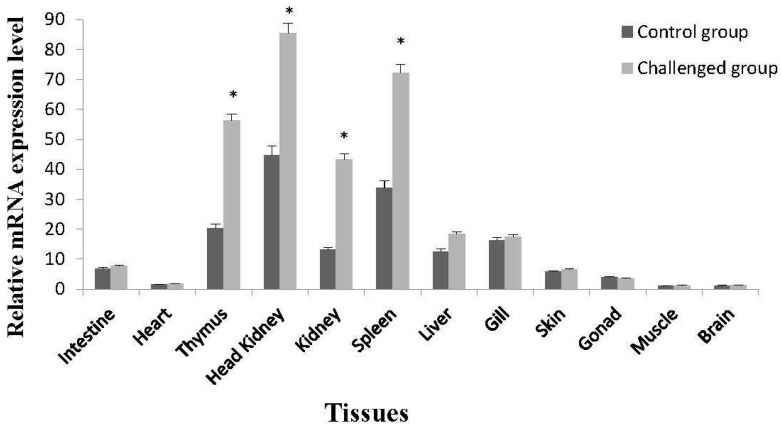
*On-mIgD* mRNA expression levels in different tissues of healthy and *S. agalactiae*-challenged *O. niloticus* measured by fluorescent real-time RT-PCR. Significant difference is indicated by asterisks. *: 0.01 < *p* < 0.05.

Tissue-specific expression characteristics of the *On-mIgD* mRNA were tested by real-time quantitative PCR (RT-qPCR). In healthy Nile tilapia, the *On-mIgD* mRNA expression could be detected in all studied tissues and with higher levels in intestine, thymus, head kidney, kidney, spleen, liver, gill, and skin (Figure 5). After challenged with *S. agalactiae* 48 h later, the *On-mIgD* mRNA expression was significantly upregulated in head kidney, thymus, and spleen, whereas the *On-mIgD* mRNA was still dominantly expressed in the tissues of kidney and liver (Figure 5).

### 2.4. Temporal Expression Profile of On-mIgD mRNA

**Figure 6:**
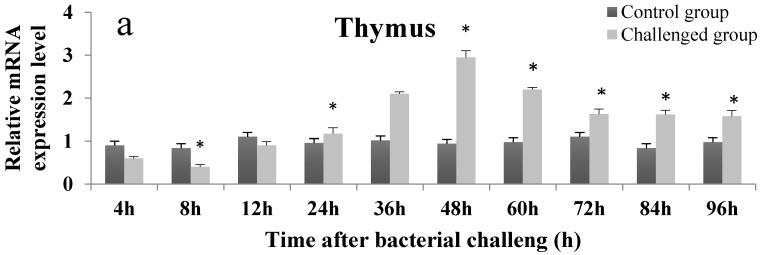

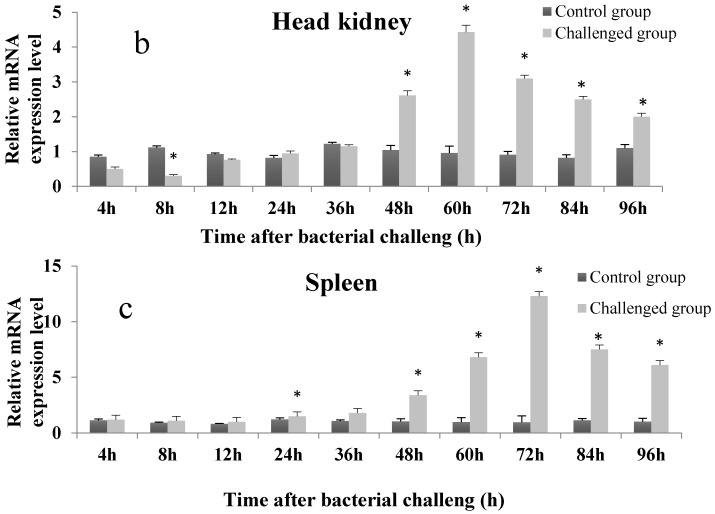
Temporal expression of *On-mIgD* in thymus (**a**); head kidney; (**b**) and spleen (**c**) of *O. niloticus* after *S. agalactiae* challenge were measured by fluorescent real-time RT-PCR. Significant difference was indicated by asterisks, *: 0.01 < *p* < 0.05.

RT-qPCR was used to examine the differential expression of *On-mIgD* mRNA. A clear time-dependent expression pattern of *On-mIgD* was observed in head kidney, thymus, and spleen in Figure 6. At 0–36 h after *S. agalactiae* challenge, the expression of *On-mIgD* mRNA was upregulated gradually and reached the maximum level at 48 h post-challenge in the thymus and head kidney, and 72 h in the spleen, respectively. As time progresses, the *On-mIgD* mRNA expression dropped gradually. According to all quantitative data, which were presented as the means ± standard deviation (SD), the *On-mIgD* expression levels were significantly higher than control levels at the time point of 48 h in the thymus, 60 h in the head kidney, and 72 h in the spleen after immunization (*p*-value less than 0.05 was considered to be significant).

## 3. Discussion

In order to understand the mechanism, evolution, and diversity of the fish humoral immune system, we need to know the genes and structure of immunoglobulins. Researchers have cloned and characterized several fish Ig cDNAs in previous studies, and discovered two different types of Igs (IgM and IgD) because of their different heavy chains. Although cDNA sequences are available for the IgDs of at least eight fish, there were no reports indicating that the full sequences, molecular features, and tissue distribution of IgD from Nile tilapia compared with those of vertebrates.

In the present study, we had determined the complete cDNA encoding of IgD heavy chains (designated *On-mIgD*) from the GIFT strain of Nile tilapia *O. niloticus* by EST and RACE techniques. Sequence analysis indicated that *On-mIgD* contained important elements which were required for a protein to be defined as an IgD family member (Figure 1 and Figure 3). The BLAST analysis and multiple sequence alignment of On-mIgD revealed that the overall sequence homology of the IgD proteins from different species was high, and shows a similar structural characteristic. A rearranged VDJ domain, a μ1 domain, 7 δ domains (δ1-δ2-δ3-δ4-δ5-δ6-δ7), and transmembrane regions compose a puzzling molecule. Yet, different fishes have different numbers of δ domains. For example, Atlantic cod comprised double domains of δ1 and δ2, while lacking domains of δ3–δ6 [3]. In contrast, Atlantic salmon [2], halibut [11], and channel catfish [13] comprised duplicated δ2-δ3-δ4-δ2-δ3-δ4 domains. Interestingly, the constant region of the fugu *IgD* gene have six δ domains, which were an extraordinary large duplication [8]. In this study, we have shown that seven δ domains occurred in the constant region of the Nile tilapia *IgD* gene, which the same as Japanese flounder IgD [4]. 

There were two forms of IgD in mammals and teleosts, which were the secreted and membrane-bound forms. So far, the secretory type of IgD has been reported in catfish, rainbow trou, and fugu [1,7,8]. According to the structure of On-mIgD, we did not find a domain located between the δ7 and the TM domains, because of the secretory forms of other species’ IgDs usually have a domain located at upstream of the transmembrane domain [8]. Moreover, different genes encoded the membrane and secretory forms of IgD [12]. Thus, whether different genes depend on the fish species encoding different forms of IgD heavy chains requires further investigation.

In order to study the functions of On-mIgD, we investigated the tissue-specific and response to inactivation of the *S. agalactiae* expression pattern. Previously, studies revealed a distribution of IgD from fugu (*Takifugu rubripes*) [8] and Japanese flounder (*Paralichthys olivaceus*) [4] throughout the spleen and head kidney. In Atlantic cod [18] and mandarin fish (*Siniperca chuatsi*) [19], IgD was highly expression in the head kidney and spleen, as well. In Nile tilapia, *On-mIgD* mRNA was highly expressed in the head kidney, following the spleen, thymus, and kidney (Figure 5). Histological analyses in teleosts show the same pattern of expression with lymphopoiesis. The head kidney of teleosts is a major immune organ and plays an important role in the innate immune system [20]. The spleen used a uniquely organized way to combine the innate and adaptive immune systems. The thymus is thought to be the primary site for functional T-cell development [21,22]. According to the data of Figure 2, Figure 3 and Figure 4, the structure of IgD is consistent with mandarin fish (*S. chuatsi*), and their sequences of amino acids have high identity as well, so the Nile tilapia and mandarin fish were highly expressed in the head kidney. The high expression level of IgD in fugu (*T. rubripes*) spleen is due to the difference in structure of IgD and the amino acid sequence with other teleosts.

Until now, little data has described the expression profile of *On-mIgD* during *S. agalactiae* challenge. In the present study we detected the *On-mIgD* expression after stimulation of inactivated *S. agalactiae*, the *On-mIgD* was constitutively expressed in the head kidney, thymus, and spleen, and a clear time-dependent pattern of *On-mIgD* expression was observed in above tissues. Interestingly, after vaccination of Nile tilapia with inactivated *S. agalactiae*, in the immune organs, such as the thymus, head kidney, and spleen, the mRNA relative expression of *On-mIgD* was downregulated from 0–12 h, implying a possible suppression of immune response in the early period of bacterial infection [23]. At 12–36 h post-challenge, the expression of *On-mIgD* mRNA was up-regulated gradually and reached the maximum level at 48 h in the thymus, 60 h in the head kidney, and 72 h in the spleen, respectively. The expression profile of *On-mIgD* has some difference compared to *On-CD2BP2*, which plays a crucial role in CD2-triggered T cell activation and nuclear splicing. Both of the times *On-mIgD* up-regulated and reached the maximum level were later than that of *On-CD2BP2*, which would have meant that T cell activation can enhance the B-cell matured in teleosts. The result shows that the expression of *On-mIgD* mRNA in the thymus and spleen were a significant rise after injection of inactivated *S. agalactiae*, and it means tilapia may fight against the bacteria by activating B and T-cell immunity after immunization [24,25]. It has been shown that *S. agalactiae* can survive inside a macrophage, which may activate cytokines associated with cellular immunity in the thymus, the site of T-cell maturation [26,27,28]. In mammals, immunoglobulin in the blood plays a critical role in modulation of the cellular immune system [29], and high similarity of the immunoglobulin between fish and mammals implies this ability may be conserved in fish.

## 4. Materials and Methods

### 4.1. Animals and Preparation

Nile tilapia, *O. niloticus* (400 g mean body weight) was obtained from a local commercial farm in Zhanjiang, Guangdong province, China. The fish were acclimated in fiber-reinforced plastic tanks (1000 L each) with aerated fresh water at 27 °C. The fish were anesthetized using MS222 before killing. We sampled twelve kinds of tissues including the skin, intestine, heart, liver, spleen, gill, head kidney, kidney, thymus, gonad, brain, and muscle from the killed tilapia and put in liquid nitrogen and stored at −80 °C for ready to use.

### 4.2. Amplification of IgD cDNA

Total RNA from the spleen was extracted using Trizol Reagent (Invitrogen, Carlsbad, CA, USA) as described in the manufacturer’s instructions, and the quality of total RNA was detected by electrophoresis on 1% agarose gel. In order to obtain the full length cDNA, degenerate primers (DF1/DR1 in Table 1) were designed based on the conserved sequences obtained from *Siniperca chuatsi* (GenBank accession no: ACO88906.1), *Epinephelus coioides* (GenBank accession no: AFI33218.1), *Paralichthys olivaceus* (GenBank accession no: BAB41204.1), and so on. Then, 5′ and 3′ ends of IgD cDNA were cloned with 5′/3′ RACE (Rapid Amplification of cDNA End) kit (Roche, Basel, Switzerland) following the manufacturer's protocol.

### 4.3. 3′ RACE

In order to obtain the 3′ end of *On-mIgD*, reverse transcriptase M-MLV (TaKaRa, Tokyo, Japan) and an oligo dT-anchor primer (Table 1) were used to synthesize the first-stand cDNA from the total RNA according to the instructions. PCR reaction was performed using an anchor primer, sense primer DR1, and a specific forward primer DF1 (Table 1). The temperature profile was 94 °C for 5 min followed by 30 cycles of 94 °C for 30 s, 56 °C for 45 s, and 72 °C for 2 min. After the final cycle, samples were incubated for a further 7 min at 72 °C for the final extension. The pMD18-T vector (Takara) was ligated with the purified PCR products, and then transformed into competent *Escherichia coli* cells. The positive clones were sequenced by Beijing Genomics Institute (Beijing, China).

### 4.4. 5′ RACE

Using previous RNA samples, cDNA was synthesized by a specific reverse primer DSP1 (Table 1), designed based on the 3′ end of the *On-mIgD* cDNA sequence. The PCR product was purified with the QIAquick PCR Purification kit. Poly A was added by using a terminal transferase (Roche) at 37 °C for 2 h. For the amplification of the 5′ end of *On-mIgD*, nested-PCR amplification was performed according to our previous method [30]. 

### 4.5. Bioinformations Analysis

The homologous analysis of the nucleotide and amino acid sequences were conducted by BLAST software (http://blast.ncbi.nlm.nih.gov/Blast.cgi). The Clustalw2 software (http://www.ebi.ac.uk/Tools/clustalw2) was used to analyze the multiple alignment of On-mIgD amino acid sequences and optimized manually. The protein motif features were conducted with ExPASy tools (http://www.ebi.ac.uk/Tools/pfa/iprscan/). MEGA4 software was used to construct a phylogenetic tree by the Neighbor-Joining method.

### 4.6. S. agalactiae Challenge of the Fish

*S. agalactiae* used for immune stimulus was isolated from *O. niloticus* [17]. The challenge experiment was performed as follows: each fish of the experimental group was injected intraperitoneally (i.p.) with 100 μL live *S. agalactiae* which was resuspended in PBS (phosphate buffer saline) (1× 10^8^ CFU/mL) and the control group was injected with 100 μL PBS. At each time point (0, 4, 8, 12, 24, 36, 48, 60, 72, 84, and 96 h), thymus, head kidney, and spleen were obtained from the control groups and treatment groups. For tissue-specific expression analysis, different tissues (skin, intestine, liver, spleen, gill, head kidney, kidney, thymus, muscle, gonad, brain, and heart) of healthy and *S. agalactiae*-challenged Nile tilapia (*O. niloticus*) were sampled at 48 h post-challenge. We sampled three individuals’ tissues from *O. niloticus* at each time point and put them in one pool as a sample.

### 4.7. Tissue-Specific Expression of On-mIgD mRNA

The before- and after-immunized tissues at 48 h, and gene-specific primers (Table 1), were used as the sample to deetect the differential expression levels of *On-mIgD* by RT-qPCR. According to the manufacture’s protocol, we used Reverse Transcriptase M-MLV (TaKaRa) to synthesize the first-strand cDNA from total RNA treated by DNase.

QrD-F and QrD-R, the two *On-mIgD* specific primers (Table 1), were used to detect the expression characteristic of *On-mIgD.* The relative expression levels of *On-mIgD* were calculated using β-actin as a reference. The primers (Table 1) designed based on sequences from GenBank (Nucleotides Accession No: ABN58893.1).

The PCR was performed in a 20 μL reaction volume containing 10 μL of 2× TransStart^TM^ Green qPCR SuperMix (TransGen, Beijing, China), 0.4 μL of each primers (10 μM), 2 μL of 10^−1^ diluted cDNA, and 7.2 μL of PCR-grade water. The PCR amplification procedure according to our previous method except for annealing temperature was 60 °C [30]. The sample was run in triplicate on the Bio-Rad iQ5 Real-time PCR System (Bio-Rad, Hercules, CA, USA). After the PCR program, fluorescent real-time PCR data were analyzed with iQ™5 Optical System Software v 2.0 (Bio-Rad). PCR efficiency was calculated according to the protocol by Schmittgen and Livak (2001) [31]. The comparative *C*_t_ method was used to analyze the expression level of *On-mIgD* [31]. The assay was performed three times.

### 4.8. The Temporal Expression of On-mIgD mRNA in Head Kidney, Thymus, and Spleen after Bacterial Challenge

The temporal expression pattern of *On-mIgD* mRNA in the head kidney, thymus, and spleen after bacterial challenge was also measured by fluorescent real-time RT-PCR. *S. agalactiae* challenge and head kidney, thymus, and spleen collecting were performed as described in Section 4.6. The differential expression levels of *On-mIgD* in pre- and post-immunized tissues at different times were measured as described above.

### 4.9. Statistical Analysis

All quantitative data were presented as the means ± standard deviation (SD). Statistical analysis was performed using SPSS statistics 17.0 software. A *p*-value less than 0.05 was considered to be significant. 

## 5. Conclusions

In summary, an IgD heavy chain was cloned from the GIFT strain of Nile Tilapia (*O. niloticus*). qRT-PCR analysis revealed that On-mIgD was expressed strongly in immune organs and responded to *S. agalactiae* infection. Our finding is expected to contribute to providing some reference for exploration of Ig-mediated innate immunity in teleosts and warrant further investigation to distinguish and understand the mechanisms of IgD.

## Figures and Tables

**Table 1:** Sequences of primers used in this study.

Primers	Sequence (5′–3′)
Oligo dT30-anchor	AAGCAGTGGTATCAACGCAGAGTACT(30)VN
DF1	GGAGTCAGTCAARTCCRAGT
DR1	CTGCTYAGRCTGAASGTTTT
DSP1	CGTTGTTTTGATTCAGTGGTTGT
DSP2	AGATGAGCCTTACTGCTGAGATT
DSP3	AAGCAGTGGTATCAACGCAGAGT
Oligo dT-anchor	GACCACGCGTATCGATGTCGACT(16) V
Anchor primer	GACCACGCGTATCGATGTCGAC
QrD-F	AACACCACCCTGTCCCTGAAT
QrD-R	GGGTGAAAACCACATTCCAGC
β*-action*-F	AACAACCACACACCACACATTTC
β*-action*-R	TGTCTCCTTCATCGTTCCAGTTT
